# Cortical thinning and associated connectivity changes in patients with anorexia nervosa

**DOI:** 10.1038/s41398-021-01237-6

**Published:** 2021-02-04

**Authors:** Feliberto de la Cruz, Andy Schumann, Stefanie Suttkus, Nadin Helbing, Regine Zopf, Karl-Jürgen Bär

**Affiliations:** 1 Lab for Autonomic Neuroscience, Imaging and Cognition (LANIC), Department of Psychosomatic Medicine and Psychotherapy, Jena University Hospital, Jena, Germany; 2 Department of Cognitive Science, Perception in Action Research Centre, Faculty of Medical, Health & Human Sciences, Macquarie University, Sydney, NSW Australia

**Keywords:** Psychiatric disorders, Neuroscience

## Abstract

Structural brain abnormalities are a consistent finding in anorexia nervosa (AN) and proposed as a state biomarker of the disorder. Yet little is known about how regional structural changes affect intrinsic resting-state functional brain connectivity (rsFC). Using a cross-sectional, multimodal imaging approach, we investigated the association between regional cortical thickness abnormalities and rsFC in AN. Twenty-two acute AN patients and twenty-six age- and gender-matched healthy controls underwent a resting-state functional magnetic resonance imaging scan and cognitive tests. We performed group comparisons of whole-brain cortical thickness, seed-based rsFC, and network-based statistical (NBS) analyses. AN patients showed cortical thinning in the precuneus and inferior parietal lobules, regions involved in visuospatial memory and imagery. Cortical thickness in the precuneus correlated with nutritional state and cognitive functions in AN, strengthening the evidence for a critical role of this region in the disorder. Cortical thinning was accompanied by functional connectivity reductions in major brain networks, namely default mode, sensorimotor and visual networks. Similar to the seed-based approach, the NBS analysis revealed a single network of reduced functional connectivity in patients, comprising mainly sensorimotor- occipital regions. Our findings provide evidence that structural and functional brain abnormalities in AN are confined to specific regions and networks involved in visuospatial and somatosensory processing. We show that structural changes of the precuneus are linked to nutritional and functional states in AN, and future longitudinal research should assess how precuneus changes might be related to the evolution of the disorder.

## Introduction

Anorexia nervosa (AN) is a severe mental disorder characterized by a distorted body image and restriction of food intake. The extreme underweight of AN patients is linked to the elevated mortality rate known to be the highest among mental disorders^1,2^. Notably, the resulting damage to the cardiovascular system contributes to elevated cardiac mortality^3,4^.

Early post-mortem investigation have related malnutrition in AN patients to changes in cerebral structures^5,6^. Modern neuroimaging studies have also provided substantial evidence for changes in brain structure in AN^7–10^. For instance, meta-analyses have revealed a reduction of gray matter volume by 4.6% and white matter volume by 2.7% in AN patients compared to healthy subjects^11,12^. Additionally, decreased cortical thickness (CT) is a consistent finding in adult and adolescent patients with AN^13,14^. After weight gain, this cortical thinning can be reversed to a normal level^15–17^. Cortical thinning seems to be confined to the somatosensory cortex, the cingulo-parietal network, and the occipital cortex^8,13,18–20^. Interestingly, decreases of CT can be detected in healthy subjects with subclinical traits for disordered eating behavior^21^. Regional cortical thinning patterns might, therefore, be an adequate marker for progression and remission of AN^20^.

It appears self-evident that structural changes in brain areas influence perceptual and cognitive function in these regions. McCormick et al. demonstrated that reduced volume of the anterior cingulate cortex relates to cognitive deficits in perceptual organization and conceptual reasoning^22^. The somatosensory cortex and superior parietal cortex are critical regions for perception and integration of bodily stimuli^23,24^. In a previous study, we showed that structural aberrations in the parietal-cingulate network are associated with alterations in thermal pain perception^25^. Joos et al. found that the extent of gray matter loss, especially in the cingulate cortex, temporoparietal regions and the precuneus, correlated with an increased desire for thinness in AN patients^26^. However, there is a need to investigate the functional consequences of structural changes in AN in more detail.

Regional changes of the cortical structure of a specific brain region are thought to be accompanied by altered functional connectivity to other brain regions^27–29^ and might, therefore, influence functional brain networks beyond those areas with structural changes. A meta-analysis revealed that AN patients exhibit altered connectivity in brain networks involved in cognitive control as well as in visuospatial and body-signal integration, including the frontal, parietal, somatosensory, cingulate, insular and occipital cortices (see review^30^). Furthermore, Favaro et al. demonstrated disrupted resting-state connectivity within the visuospatial and somatosensory networks and correlation of the latter with lower visuospatial abilities in AN patients^31^.

However, until now, structural and functional brain changes in AN patients have been mostly analyzed separately (e.g. see review^32^). Therefore, it needs to be determined how the well-documented changes of cortical thickness might relate to alterations in functional brain organization^20^. We hypothesized that cortical abnormalities in brain regions putatively involved in the pathophysiology of AN are closely linked to brain function disturbance. Thus, using a cross-sectional, multi-modal imaging approach, we analyzed alterations of resting-state functional connectivity in those regions affected by structural changes in AN patients.

## Materials and methods

### Participants

We recruited twenty-seven patients with AN and twenty-six age- and gender-matched healthy controls (HC). A research psychiatrist assessed inclusion and exclusion criteria for patients and controls. To include patients, they had to meet the DSM-IV criteria for anorexia nervosa according to the Structured Clinical Interview for DSM-IV Axis I disorders. Patients were recruited from the specialized ward for eating disorders at our institution and investigated within the 3^rd^ and 7^th^ days after admission. This period allows patients to acclimate to the hospital environment and reduces interferences from pharmacological or psychotherapeutic interventions.

Further inclusion criteria for HC and AN were right-handedness as evaluated by the modified version of Annett’s handedness inventory^33^ as well as an age range of 18 to 50 years. All patients and controls were thoroughly examined by means of a clinical examination, standard electrocardiogram and routine laboratory investigations. In addition, patients and controls were screened for a history of other Axis 1 or Axis 2 disorders such as major depression, personality disorders or obsessive compulsive disorder. Specific attention was paid on somatic conditions due to malnutrition, e.g. cardiac conditions. Five patients were excluded due to an interfering psychiatric or somatic condition mentioned above. According to the SCID-I interview^34^, none of the HC had a current episode or a history of a mental disorder. For HC, we also asked for first-degree relatives with a psychiatric disorder and only included those who had no first-degree relatives with a diagnosis. All participants had normal or corrected-to-normal vision, according to the information provided by them.

Informed written consent was obtained from all participants one day in advance before entering into the study following the protocols approved by the local Ethics Committee of the Friedrich-Schiller University. Table 1 shows a full description of participant characteristics.

**Table 1:** Demographics data and neuropsychological test performance.

	Patients	Controls	Significance^a^
*Parameter*			
Age	23.8 ± 7.2	25.2 ± 6.6	n.s.
Gender (female/male)	19/3	24/2	n.s.
BMI	15.1 ± 1.4	24 ± 3.2	*p* < 0.001
*Education*			
No	*n* = 1	*n* = 0	
Primary	*n* = 0	*n* = 0	
Secondary	*n* = 19	*n* = 26	
n.a.	*n* = 2		
*Eating disorder inventory scores (EDI-2)*			
Drive for thinness	27.2 ± 6.7	16.9 ± 6	*p* < 0.001
Bulimia	20.7 ± 10.4	11.3 ± 5.9	*p* < 0.001
Body dissatisfaction	32.7 ± 9.3	27.9 ± 9.6	n.s.
Ineffectiveness	36.1 ± 8.1	23.9 ± 7.6	*p* < 0.001
Perfectionism	19.3 ± 5.9	16.5 ± 4.5	n.s.
Interpersonal distrust	25.1 ± 5.7	17.9 ± 3.8	*p* < 0.001
Interoceptive awareness	34.5 ± 8.4	19.9 ± 7.1	*p* < 0.001
Maturity fears	26.8 ± 5.5	24.6 ± 6.8	n.s.
Overall EDI score	222.2 ± 39.5	158.8 ± 31.3	*p* < 0.001
*Anxiety and depressive symptoms*			
STAI trait	48.5 ± 9.0	36.3 ± 8.7	*p* < 0.001
STAI state	51.5 ± 11.1	37.3 ± 6.7	*p* < 0.001
BDI	22.7 ± 8.6	6.2 ± 5.1	*p* < 0.001
*Cognitive performance*			
TMT A [ms]	24.8 ± 8.4	26.5 ± 7.3	n.s.
TMT B [ms]	53.3 ± 16.1	51.4 ± 16.9	n.s.
IQ (MWTB)	103.7 ± 13.8	107 ± 15.4	n.s.

^a^*t*-test; *n.s.* not significant, *BMI* body mass index, *n.a.* not accessible data, *STAI* State-Trait Anxiety Inventory, ms milliseconds, *TMT* Trail Making Test, *MWTB* Multiple Choice Word Test.

### Cognitive testing and psychopathology

Participants completed a cognitive test battery to asses premorbid intelligence (Multiple Choice Word Test; MWT-B), attention (Trail Making Test-A; TMT-A) and executive functioning (TMT-B). Moreover, general psychopathology was assessed using the Eating Disorder Inventory-2 (EDI-2), the State-Trait Anxiety Inventory (STAI trait, STAI state) as well as the Beck Depression Inventory (BDI-2) self-report questionnaire. The BDI-II^35^ is a 21-question self-report inventory, assessing the somatic, cognitive and affective symptoms of depression in the preceding two weeks. BDI-II items are rated on four-point scales ranging from 0 to 3 with a maximum total score of 63. Scores between 29 and 63 might indicate a severe depressive episode.

The MWT-B^36^ consists of 37 items in ascending difficulty each requiring identification of one truly existing word opposed to three distractors. It has been shown to be an economic, easy to administer and robust estimate of global crystallized intelligence. The TMT-A^37^ requires an individual to draw lines sequentially connecting 25 encircled numbers distributed on a sheet of paper. Task requirements are similar for TMT-B (Army Individual Test Battery, 1944) except the person must alternate between numbers and letters (e.g., 1, A, 2, B, 3, C, etc.). The score on each part represents the amount of time required to complete the task. The EDI-2^38^ evaluates symptoms and psychopathologic features of Eating Disorders. It consists of 91 items subdivided into 11 subscales. It supplies a psychopathologic profile that can be compared to the normal one. The purpose of the STAI^39^ is to measure the presence and severity of current symptoms of anxiety as well as a general tendency to be anxious. There are two subscales within this measure. The state scale evaluates the current state of anxiety, asking how individuals feel right now. The trait scale evaluates relatively stable aspects of anxiety proneness. The STAI has 40 items, 20 items allocated to each of the two subscales.

#### fMRI data acquisition

Data were collected on a 3 T whole body-system equipped with a 12-element head matrix coil (MAGNETOM Prisma, Siemens Healthcare, Erlangen, Germany). The protocol consisted of a resting state scan, followed by a structural scan. We instructed participants to keep their eyes open to avoid that some may fall asleep and so reduce inter-individual variability in wakefulness states^40^. T_2_*-weighted images were obtained using a multiband multislice GE-EPI sequence (TR = 484 ms, TE = 30 ms, flip angle = 90°, multiband factor = 8) with 56 contiguous transverse slices of 2.5 mm thickness covering the entire brain. The matrix size was 78 × 78 pixels with an in-plane resolution of 2.5 × 2.5 mm^2^ corresponding to a field of view (FOV) of 195 mm × 195 mm. A series of 1900 whole-brain volume sets were acquired in one session lasting approximately 15 min.

High-resolution anatomical T_1_-weighted volume scans (MP-RAGE) were obtained in sagittal orientation (TR = 2300 ms, TE = 3.03 ms, TI = 900 ms, flip angle = 9°, FOV = 256 mm×256 mm, matrix 256 × 256, number of sagittal slices = 192, parallel acquisition technique (PAT) factor = 2 with an isotropic resolution of (1 × 1 × 1) mm^3^.

#### Physiological recordings and analyses

Cardiac and respiratory activities were recorded (500 Hz) during rs-fMRI data acquisition using an MR-compatible BIOPAC MP150 polygraph (BIOPAC Systems Inc., Goleta, CA, USA). Respiratory activity was assessed by a strain gauge transducer incorporated in a belt tied around the chest, approximately at the level of the processus xiphoideus. The cardiac signal was recorded using a standard photoplethysmograph (PPG) attached to the proximal phalanx of the index finger of the subject’s left hand. The PPG measures blood volume change in the microvascular bed of tissue by measuring the varying intensity of light traveling through the tissue^41^. The PPG can be used as a surrogate technique for the electrocardiogram (ECG) and is usually preferred to ECG systems for cardiac recordings since ECG-derived signals exhibit greater susceptibility to electromagnetic and biologic interference^42^. PPG and respiratory signals were digitally filtered at 0.05–3 Hz, 0–10 Hz, respectively, to remove MRI-related or movement artifacts.

#### Resting-state fMRI preprocessing

Data preprocessing was performed using the “afni_proc.py” script in the AFNI software package (https://afni.nimh.nih.gov/). After discarding the first twenty volumes to allow for magnetic saturation, we identified volumes with excess motion (Euclidean norm of the motion derivatives > 0.3 mm or fraction of voxel outliers > 10%) and removed signal spikes in the signal intensity time courses. Next, artifacts time-locked to the cardiac and respiratory cycles as well as slow blood oxygenation level fluctuations were modeled via RETROICOR^43^ and respiration volumes per time (RVT) regressors, respectively^44,45^. The model included eight RETROICOR regressors (four respiratory and four cardiac) and five RVT functions delayed at 0, 5, 10, 15, and 20 s^44^. All regressors were generated on a slice-wise basis by AFNI’s RetroTS.m script^46^.

Further preprocessing included alignment of each EPI volume to the volume with minimum outlier fraction, spatial registration of the aligned time series data to the anatomical scan, and warping of the anatomical scan to Montreal Neurological Institute (MNI) template. This transformation was also applied to the functional data, which were subsequently smoothed with a 6-mm full-width half-maximum (FWHM) Gaussian kernel. Volumes with excessive motion were censored, and subjects with greater than 20% of volumes corrupted were excluded from further analysis^47^. One patient exceeded this threshold and was discarded for resting-state functional connectivity (rsFC) analysis. Additionally, functional data were bandpass filtered to retain frequencies between 0.01–0.1 Hz and contributions of non-neural sources were reduced by regressing the following nuisance variables: (1) 12 motion regressors (6 realignment parameters and their derivatives), (2) voxelwise local white matter regressors, and (3) 3 principal components of ventricle signals^46^. Masks for white matter and ventricles were generated from each participant’s anatomical scan using Freesurfer 6.0.0 (http://surfer.nmr.mgh.harvard.edu). Censoring, nuisance regression, and bandpass filtering were performed in a single step.

### Cortical thickness and functional connectivity

We used FreeSurfer’s automated segmentation pipeline to obtain reliable estimates of cortical thickness. This segmentation process has been described in detail elsewhere^48,49^. Briefly, it performs the following steps (1) removal of non-brain tissue, (2) registration to the MNI space, (3) intensity inhomogeneity correction, (4) tissue-type classification, (5) and probabilistic anatomical labeling. Cortical thickness (CT) is computed by finding the shortest distance between a given point on the estimated pial surface and the gray/white matter boundary and vice versa and averaging these two values.

We conducted whole-brain vertex-wise group comparisons of CT between HC and AN, controlling for age as a nuisance covariate. To this end, subject CT maps of the left and right hemispheres were separately mapped onto the FreeSurfer “faverage” surface and smoothed using a Gaussian kernel with an FWHM of 10 mm. A Monte Carlo simulation cluster analysis (10 000 iterations) was then performed to correct for multiple comparisons using an initial cluster-forming threshold *p* < 0.01 and cluster-wise threshold *p* < 0.01 with Bonferroni correction to account for both hemispheres.

Statistically significant clusters of CT were used as seed regions for subsequent rsFC analysis. The resulting rsFC maps were transformed to Z maps using Fisher’s Z transformation^50^ and compared between groups. The AFNI’s *3dClustSim* program was used to correct for multiple comparisons, a minimum clustersize threshold of 89 voxels was necessary for identifying significant differences at α < 0.05 with an initial voxelwise threshold of *p* < 0.005.

### Network-based statistical analysis

In addition to the seed-based rsFC approach, we investigated significant between-group differences in the whole-brain network connectivity using the network-based statistic (NBS) framework^51^. NBS is a validated non-parametric method used to control the family-wise error rate (FWER) when performing mass univariate testing on all connections in a network. The main goal of using NBS in our study was to identify potential differences in functional connectivity not accounted for the pre-defined seed regions.

Individual connectivity matrices were generated extracting the mean time series from 264 independent anatomical regions-of-interest (ROIs), which were defined based on the coordinates from an extensively validated parcellation system provided by^52^. Each ROI was modeled as a 10 mm diameter sphere with a minimum distance of 10 mm between sphere centers to avoiding overlapping. We discarded short-distance correlations less than 20 mm as they might be affected by spatial smoothing or reslicing. Components, or subnetworks, were identified using a primary component-forming threshold at *t* > 4.5. Permutation testing (10,000 permutations) was then applied to determine an empirical null distribution of maximal component sizes and assigns an FWE-corrected p-value to each component. Subnetworks with corrected *p* < 0.01 were considered as statistically significant.

## Results

### Cortical thickness

**Fig. 1:**
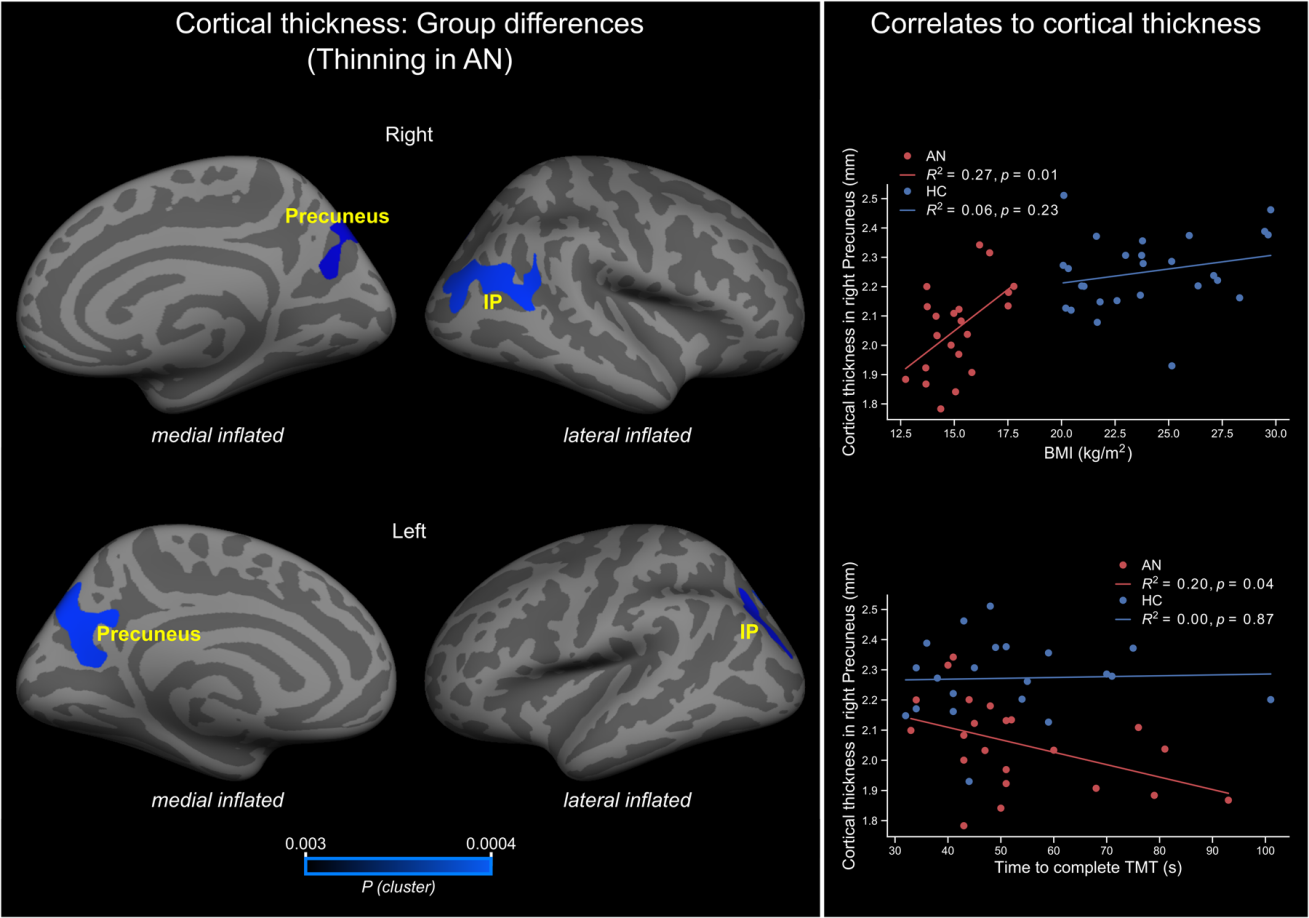
Whole-brain group differences in cortical thickness and correlation with BMI and TMT-B. Left, brain regions showing significantly cortical thickness differences between AN and healthy controls. Right, correlation between cortical thickness in the right precuneus with BMI (top) and time to complete TMT-B (bottom). Colorbar shows cluster-level family-wise corrected *p*-values (CWP). Abbreviations: IP = inferior parietal; AN = anorexia nervosa; HC = healthy controls; *R*^2^ = variance explained; *p* = *p*-value; BMI = body mass index; TMT = Trail Making Test.

We observed significantly reduced cortical thickness in AN patients compared to HC in the precuneus and inferior parietal regions (Fig. 1, left and Table 2). The differences in cortical thickness remained significant, even after controlling for age. No significantly increased cortical thickness was observed in AN patients compared with healthy controls.

**Table 2:** Regions where cortical thickness differs between AN patients and HC after controlling for age.

Region:	Hemisphere	Cluster size (mm^2^)	MNI coordinate			CWP*
			*x*	*y*	*z*	
Precuneus	R	628	18	−77	43	0.0028
Inferior parietal	R	1503	44	−60	22	0.0004
Precuneus	L	1328	−15	−63	29	0.0004
Inferior parietal	L	699	−31	−75	33	0.0008

All regions show cortical thinning in AN patients.

*Clusterwise *p*-value or *p*-value of the cluster.

### Correlation of cortical thickness with BMI and cognitive performance

In order to assess the relationship of structural changes with nutritional and cognitive states, a linear regression analysis was performed between cortical thickness and BMI and Trial Making Test (TMT). This analysis revealed a significant correlation (*R*^2^ = 0.27, *p* = 0.01 uncorrected, two-sided, Fig. 1 top right) between cortical thickness in the right precuneus (BA6/24, MNI coordinates: *x*, *y*, *z* = 18, -77, 43; cluster size = 628 mm^2^) and BMI in AN patients. Similarly, the cortical thickness in this cluster also correlated significantly (*R*^2^ = 0.20, *p* = 0.04 uncorrected, two-sided, Fig. 1 bottom right) with the time to complete TMT-B in AN patients.

#### Functional connectivity of regions with reduced cortical thickness

**Fig. 2:**
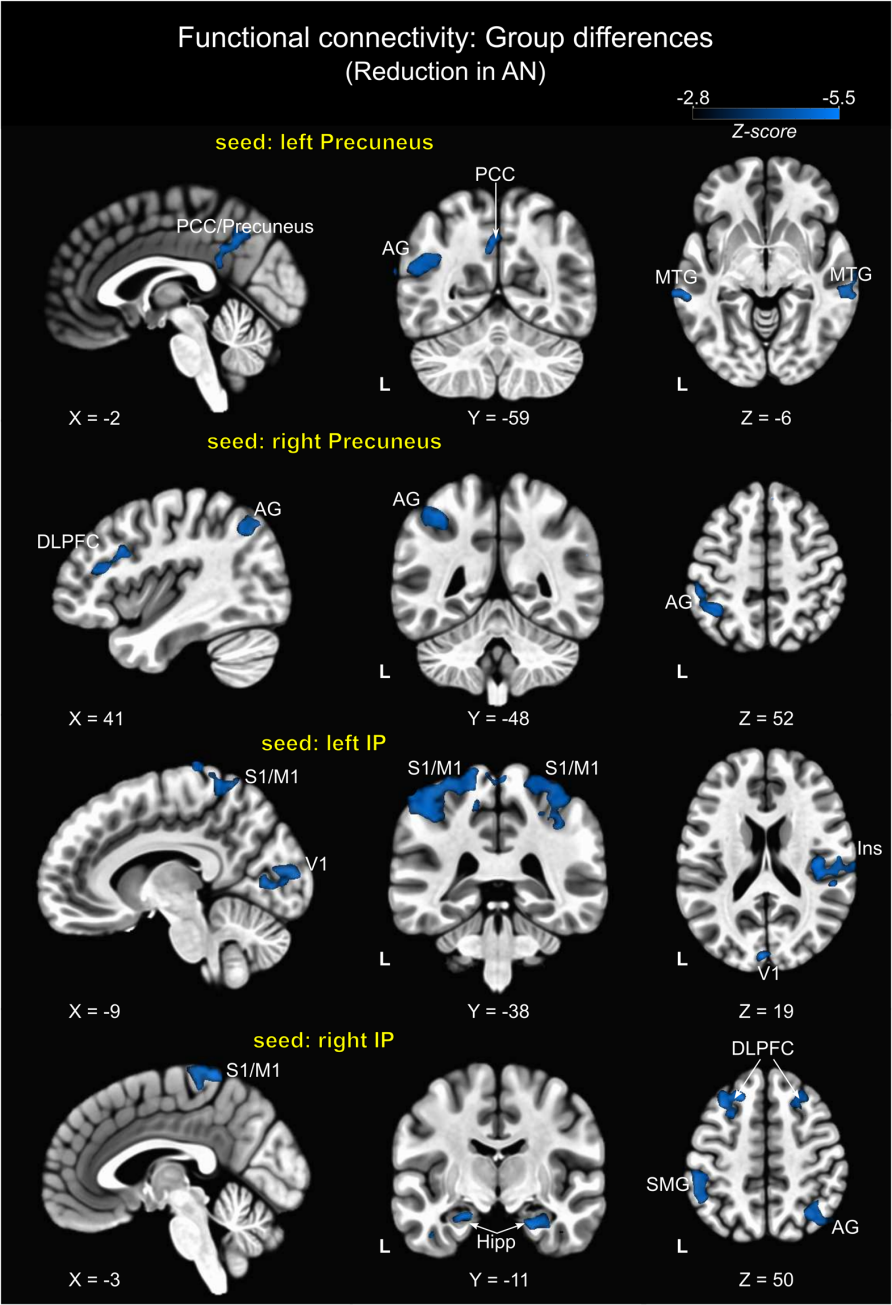
Group differences in functional connectivity using clusters of cortical thinning as seed regions. All clusters show reduced functional connectivity in AN patients. Abbreviations: AN = anorexia nervosa; IP = inferior parietal; PCC = posterior cingulate cortex; AG = angular gyrus; MTG = middle temporal gyrus; DLPFC = dorsolateral prefrontal cortex; S1/M1 = primary sensorimotor cortex; Ins = insula; Hipp = hippocampus; SMG = supramarginal gyrus.

The four clusters identified in the surface-based structural analysis showed reduced rsFC to various brain regions across major brain networks in AN patients. Compared to HC, patients did not show significantly increased rsFC in any brain region. Figure 2 and Table 3 show between-group functional connectivity differences from the seed regions: left/right precuneus, and left/right inferior parietal lobes. As shown, using precuneus as a seed region, significant rsFC differences were observed in areas of the default mode (DMN) and central executive (CEN) networks. For instance, the left precuneus showed reduced rsFC with right middle temporal (MTG) and left angular (AG) gyri as well as the precuneus/posterior cingulate cortex, which are all part of the DMN. Left and right dorsolateral prefrontal cortex (DLPFC; CEN), as well as left AG, were the regions showing between-group differences when the right precuneus was used as seed region.

**Table 3:** Brain regions showing significant differences in rsFC between AN patients and HC.

Functional connectivity to:	Right/Left	Brodmann’s area	Cluster size*	MNI coordinate			Z-value
				*x*	*y*	*z*	
Seed: left Precuneus							
PCC/Precuneus		7	154	0	−57	40	−3.6
Middle Temporal Gyrus	L	21	292	−70	−34	−2	−3.7
Middle Temporal Gyrus	R	21	145	70	−34	−2	−3.82
Angular Gyrus	L	39	181	−45	−61	28	−3.73
Seed: right Precuneus							
Angular Gyrus	R	39	213	44	−64	44	−3.35
Inferior Parietal Lobule	L	40	199	−38	−52	50	−3.95
Dorsolateral Prefrontal Cortex	R	46/9	148	42	16	28	−3.53
Dorsolateral Prefrontal Cortex	L	8	106	−33	26	48	−3.41
Seed: left Inferior Parietal							
Primary Sensorimotor Cortex	L	2/40	1084	−50	−42	53	−3.81
Primary Sensorimotor Cortex	R	2/40	577	40	−39	60	−3.59
Cuneus	L	18	316	0	−89	20	−3.73
Primary Visual Cortex	L	17		−7	−76	4	−3.31
Insula	R	13/40	254	49	−23	17	−2.8
Visual Association Cortex	R	18	234	20	−57	3	−4.11
Seed: right Inferior Parietal							
Primary Sensorimotor Cortex	L	4/6	150	−3	−42	70	−4.38
Precuneus	L	7	119	−5	−71	48	−3.89
Angular Gyrus	R	7/39	400	37	−62	47	−3.54
Supramarginal Gyrus	L	40	192	−51	−44	50	−3.64
Hippocampus	R		190	26	−11	−24	−4.12
Hippocampus	L		142	−23	−11	−19	−3.44
Dorsolateral Prefrontal Cortex	R	9	297	27	26	40	−3.85
Dorsolateral Prefrontal Cortex	L	8	237	−30	23	53	−3.81

All clusters show reduction in rsFC in patients.

*Voxel level *p* < 0.005 uncorrected, cluster level *p* < 0.05 corrected.

Using left/right IP lobes as seed regions, rsFC differences were more widespread throughout the brain and involved regions of the sensorimotor, salience, DMN and visual networks. For the left IP, rsFC differences to the left/right sensorimotor cortex (S1/M1) and left insula (salience network) were observed. Further clusters appeared in the visual network, represented by the cuneus, primary visual cortex (V1) and the visual association cortex. With respect to the right IP, overall between-group differences were detected for connectivity to the left/right DLPFC, left/right hippocampus (part of the DMN), right AG, precuneus as well as to the left sensorimotor cortex (S1/M1) (Fig. 2 and Table 3).

#### Network-based statistic

**Fig. 3:**
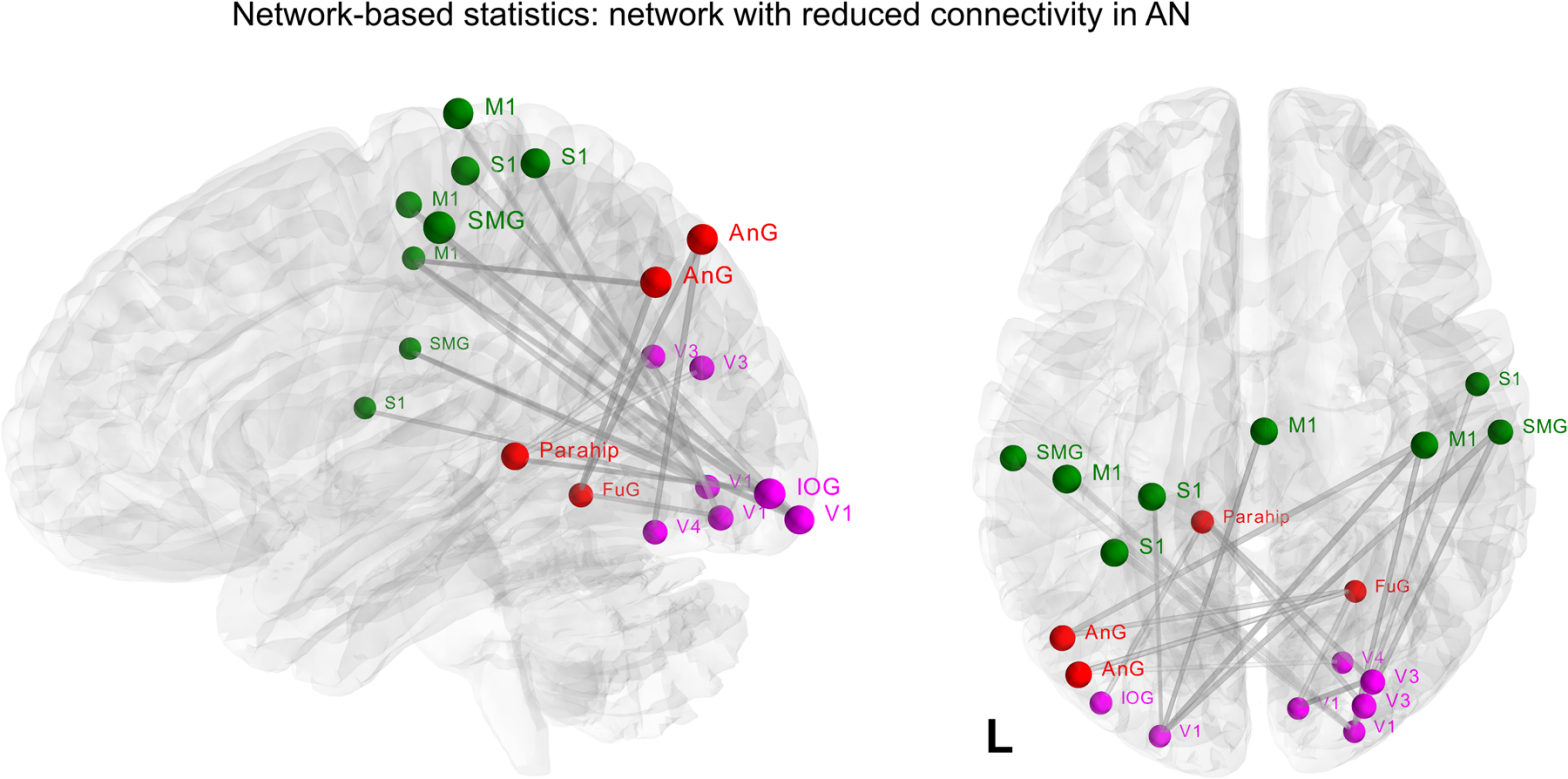
Group comparisons in functional connectivity matrices using Network-Based statistics (NBS). The depicted component shows nodes with significantly (*p* < 0.01 corrected) reduced connectivity in AN patients compared to HC. These connections formed a single connected network with 19 nodes and 18 egdes. Nodes are color-coded according to the cerebral network where they belong to. Sensorimotor network (green), default-mode network (red) and visual network (pink). Coordinates are shown in Supplementary Table S1. Abbreviations: M1, primary motor cortex; S1, primary sensory cortex; SMG, supramarginal gyrus; Ang, angular gyrus; FuG, fusiform gyrus; Parahip, parahippocampus; V1, primary visual cortex; V4, visual area 4; IOG, inferior occipital gyrus.

We used NBS analysis to identify networks that were not accounted for by the seed-based correlation analysis. Here, significantly reduced positive rsFC was observed in patients in a subnetwork of 19 nodes and 18 edges (Fig. 3: *p* < 0.01). Nodes within this network were located in sensorimotor areas mainly, i.e. S1, supramarginal gyrus and M1, but also in occipital, angular and fusiform gyri and parahippocampus regions with a large number of intra-hemispheric functional connections. These regions are part of the three major brain networks, namely sensorimotor (green), default-mode (red) and visual networks (pink).

## Discussion

Structural changes in the brain have been consistently identified in AN patients^25,32,53^. In particular, cortical thinning is one of the most frequent findings in AN, and hence, proposed as a reliable biomarker of the illness^20^. However, little is known whether this type of structural change might affect the functional coupling of brain regions. The current study shows cortical thickness abnormalities and corresponding functional connectivity alterations in acute AN patients. In line with previous works, we found cortical thinning in the precuneus and inferior parietal lobule. Both regions play a crucial role in domains affected in AN patients such as perception, visuospatial memory, and imagery and therefore implicated in the pathophysiology of the disorder^54–56^. Importantly, it seems that the precuneus and parietal lobe are responsible for the perceptive component of the body image distortion in AN according to a model proposed by Gaudio and Quattrocchi^54^. Using these brain areas as seed regions, we also revealed reductions in functional connectivity, mainly in sensorimotor, default mode, and visual networks. Functional connectivity alterations seem to be confined to these areas, as shown by the NBS analysis, which revealed a subnetwork comprising mostly sensorimotor-occipital regions.

In addition to the critical role of the precuneus in the pathophysiology of AN^57–59^, the region has also gained attention for its hub function in the overall brain network^60^. Hubs are central brain regions with high degree centrality and are relevant for efficient communication within the network^61,62^. Alterations in brain hubs are repeated findings in several neuropsychiatric disorders, including schizophrenia^63^, Alzheimer’s disease^64^, major depression disorder^65^, and others^66^. Since hubs are biologically costly, they tend to be more exposed to pathogenic processes^66^. For example, the precuneus has a high metabolic rate, consuming ~35% more glucose than any other area of the cerebral cortex in humans^67^. Therefore, it is conceivable that the precuneus is one of the first and most affected brain areas due to malnutrition. Crossley and colleagues proposed an intriguing theory that posits that lesions concentrate on hubs because of their higher topological values^66^. They argued that some disease processes lead to symptoms when the lesion occurs in a hub or that some disease processes might only become symptomatic once they have propagated to topologically central nodes. Based on this theory, one could speculate that AN might progress in a more severe stage of illness once that hub regions are affected. By contrast, AN might become more treatable if brain abnormalities shift towards non-hub nodes^66^. Indeed, recent investigations suggest that during the recovery stage, structural alterations manifest predominantly in regions topologically less complex than the precuneus, such as the cerebellum^68^, superior frontal cortex^69^, or striatum^70,71^.

Further important hints favoring the precuneus involvement in the pathophysiology of AN are the correlation of cortical thickness with BMI. Contrary to our previous publication^25^ and other studies^13,15,72,73^, we found an association between cortical thickness and BMI. Overall, the inconsistency in the relationship between structural and nutritional state indices is not well understood. The BMI during admission cannot reflect the high variability of the disease process lasting months or years with various episodes of weight gain or loss. Therefore the relation of cortical thickness and the actual BMI might be rather weak. Besides, other parameters such as illness duration, nutritional status, brain development, and hydration are confounding factors in AN neuroimaging and might affect the cortical thickness-BMI correlation^53^. Indeed, Lavagnino and colleagues suggested that the relationship between cortical thickness and BMI might be present only in an early phase of the illness, and tend to disappear in subsequent stages of the disorder due to the effects of prolonged malnutrition or compensatory mechanisms^73^.

An interesting finding of our study is the association of precuneus’ cortical thickness and TMT performance in AN patients - the thinner the precuneus cortex, the longer the time to complete TMT. TMT is a measure of cognitive flexibility, and this latter is often impaired in AN due to the self-imposed drive for perfectionism^74,75^. However, although cognitive rigidity is a characteristic psychological AN trait, relationships between structural and cognitive indices are not often reported^76^. The precuneus plays an essential role in cognitive processing (see review^67^) with structural changes associated with cognitive impairment in this region^77^. We argue that similar to the association of structural abnormalities and BMI, a correlation between cortical thickness and cognitive performance may be present during specific stages of the illness. In support of this hypothesis is the finding of an improvement of the cognitive flexibility during weight gain^78,79^, which may eventually lead to the uncoupling of structural and cognitive indices.

Nonetheless, whether structural alterations in some brain regions have causal implications on symptoms or are instead an effect of malnutrition or a combination of both is still a matter of debate and needs further research^20,53,80,81^. Current views on anorexia nervosa favor the assumption that structural changes are a consequence of severe starvation and do not influence illness symptomatology^53^. Recent research conducted in adolescents and young adults supports this opinion by showing that cortical thickness normalizes during weight recovery^15,16^. However, the question remains why structural changes are restricted to some critical brain areas only.

Moreover, in our study AN patients showed reduced functional connectivity in major brain networks when using cortical thinning areas as seed regions. The present findings constitute clear evidence of how structural alterations are linked to functional connectivity changes. Although it is well-accepted that structural brain abnormalities lead to functional disturbance^82^, it is often difficult to demonstrate such a relationship. In the reviewed AN studies, we did not find any study relating structural abnormalities to functional connectivity alterations or vice versa. This lack of research is probably associated with the fact that functional networks are not merely a one-to-one reflection of the underlying structural network^83^. Direct evidence for this phenomenon comes primarily from task-based fMRI experiments, which show that any particular task can trigger variation in functional connectivity despite an unchanged brain structure^64^.

Furthermore, our study showed reduced rsFC of both precunei with regions engaged in self-reference, including the angular gyrus and posterior cingulate cortex (the so-called default mode network)^84,85^ and other regions involved in emotional memory (i.e., middle temporal gyrus)^86^ and cognitive control (i.e., DLPFC)^87^. Results are consistent with previous works^59,88^ and may represent the reduced propensity to regulate emotionality^89^ and impulse control^90^. Structural and functional abnormalities in precuneus are thought to be related to the body image distortion experienced by AN patients^54,91,92^. Contrary to our findings, Cowdrey and colleagues found increased rsFC within the DMN in recovered patients^57^. That finding may suggest that together with cortical thickness, the rsFC of the precuneus is also illness stage-dependent and stabilizes with the weight gain^93^. In contrast, rsFC of inferior parietal lobules predominantly revealed reduced connectivity within sensorimotor, limbic, and parieto-occipital regions. These results were confirmed by NBS analysis and are in line with a previous NBS study showing visuomotor disturbance in AN^94^. However, it is worth noting that our findings are not entirely consistent with two other NBS studies that found, respectively, alteration in thalamo-insular^95^ and cerebellar-insular-parietal-cingular^96^ sub-networks. Such discrepancies can be due to differences in the methodology used (e.g. data processing and parcellation schemes)^97,98^, differences in the clinical state of the disorder^99^ or sample composition (e.g. age range and psychiatric comorbidity)^96^. Overall, our rsFC analysis based on cortical thinning shows abnormalities in networks related to the main symptom domains of AN, such as impaired cognitive control and flexibility, as well as abnormal visual and somatosensory integration^30^.

Methodologically, our study has some advantages over priors studies that strengthen the validity of the results. Our pipeline incorporates the most recent preprocessing steps recommended for the analyses of resting-state fMRI data. In particular, the application of censoring to remove volume with excess motion is highly recommended in fMRI^100^. Censoring reduces the likelihood of inflating connectivity differences between groups^101,102^. Similarly, the use of RETROICOR has become a standard step since physiological fluctuations from cardiac and respiration cycles constitute the major sources of noise in the BOLD signal^43^. Regarding this point, the repetition time (TR) used in this study alleviates the problem of aliasing commonly found in fMRI data. Although our TR of 484 ms does not resolve heart rate frequencies above 62 bpm, it is quite useful for scanning AN patients. One of the frequent symptoms of anorexia nervosa is the alteration of heart rate^103^. In addition to weight loss, lower heart rate is likely the most consistent physical finding of AN patients. Without a sufficiently short TR, one may report spurious differences between groups caused by aliasing of physiological signals.

This study has some limitations that need to be addressed. First, we acknowledge that a longitudinal study recruiting the same AN subjects may help investigate how the normalization of cortical thickness with weight restoration impacts brain functioning. At the same time, a longitudinal analysis may shed more light on the changes in the correlation between nutritional state and cortical thickness. Second, the small sample size represents another limiting factor that could undermine the statistical tests conducted throughout the study. Nevertheless, despite this limitation, we found substantial group differences in cortical thickness and corresponding functional connectivity, suggesting that both variables are reliable biomarkers of the disorder^20,30^.

## Conclusions

To conclude, our study shows that severely ill AN patients present cortical thinning in specific regions involved in the physiopathology of the illness, such as the precuneus and inferior parietal lobules. The role of precuneus in anorexia nervosa is evidenced by the positive correlation of the cortical thickness in this region with BMI and the negative correlation with cognitive functions, as assessed by TMT-B. Such structural abnormalities are accompanied by functional connectivity reductions in AN patients across major brain networks, including DMN, sensorimotor and visual networks. Based on our findings and the literature reviewed, we are convinced that the precuneus plays an essential role in the etiology of AN and the degree of damage in this region is critical to understand the evolution of the disorder.

## Supplementary information

Table S1. Network-based statistics results.
